# Linking Critical Thinking Dispositions to Well-Being in Higher Education: A Cross-Sectional Study

**DOI:** 10.3390/healthcare14040530

**Published:** 2026-02-20

**Authors:** Olga Valentim, Raquel Simões de Almeida, Rita Marques, Isabel Lucas, Leila Sales, Rita Payan-Carreira, José Lopes

**Affiliations:** 1 Nursing Research Innovation and Development Centre of Lisbon (CIDNUR), 1600-190 Lisbon, Portugal; 2 School of Nursing, Universidade de Lisboa, 1600-190 Lisboa, Portugal; 3 RISE-Health, Nursing School of Porto, 4200-319 Porto, Portugal; 4 CIR, E2S, Polytechnic of Porto, 4200-072 Porto, Portugal; afa@ess.ipp.pt; 5 Portuguese Red Cross Higher School of Health—Lisbon (ESS CVP-Lisbon), 1300-125 Lisboa, Portugal; rmarques@esscvp.eu (R.M.); ilucas@esscvp.eu (I.L.); lsales@esscvp.eu (L.S.); 6 Center for Interdisciplinary Research in Health, Universidade Católica Portuguesa (CIIS-UCP), 1649-023 Lisboa, Portugal; 7 Center for Innovative Care and Health Technology (ciTechCare), 2414-016 Leiria, Portugal; 8 Instituto de Saúde Ambiental, Universidade de Lisboa (ISAMB-UL), 1649-028 Lisboa, Portugal; 9 Health Sciences Research Unit: Nursing (UICISA: E), 3046-851 Coimbra, Portugal; 10 Comprehensive Health Research Centre (CHRC), 7002-554 Évora, Portugal; rtpayan@uevora.pt; 11 Centro de Investigação em Educação e Psicologia (CIEP), 7002-554 Évora, Portugal; 12 Department of Veterinary Medicine, Universidade de Évora, 7004-516 Évora, Portugal; 13 Department of Education and Psychology, University of Trás-os-Montes and Alto Douro, 5000-801 Vila Real, Portugal; jlopes@utad.pt; 14 Centre for Research and Intervention in Education, Faculty of Psychology and Education Sciences of the University of Porto CIIE-UP, 4200-135 Porto, Portugal

**Keywords:** critical thinking, well-being, higher education

## Abstract

**Background/Objectives:** Mental health challenges are increasingly prevalent among higher education students, with significant implications for academic success and personal development. Emerging research suggests that critical thinking dispositions may support psychological well-being by enhancing resilience and adaptive coping. This study aimed to investigate the relationship between critical thinking dispositions and psychological well-being and to identify key sociodemographic predictors in this context. **Methods:** A cross-sectional design was employed from December 2024 to May 2025, recruiting 429 students from Portuguese higher education institutions via convenience sampling. Participants completed validated self-report measures: the Critical Thinking Dispositions Scale (CTDS) and the Psychological Well-Being Scale (PWBS), assessing seven critical thinking dispositions and six well-being dimensions, respectively. Sociodemographic data were also collected. Descriptive statistics, independent *t*-tests, one-way ANOVA, Pearson correlations, and hierarchical multiple regression were used for data analysis. **Results:** Students demonstrated moderate to high levels of critical thinking and psychological well-being, with higher scores associated with increased age and academic progression. Significant positive correlations were identified between critical thinking dispositions and all well-being dimensions; personal growth, purpose in life, and autonomy exhibited the strongest associations. Regression analysis revealed that confidence in reasoning, cognitive maturity, and open-mindedness were significant predictors of psychological well-being, explaining 28.7% of the variance. Conversely, inquisitiveness showed a negative association with psychological well-being in the multivariate model, an unexpected finding that warrants cautious interpretation and further investigation. **Conclusions:** Critical thinking dispositions reflect affective tendencies and habitual ways of engaging with thinking. These dispositions appear to protect psychological well-being in higher education students. Integrating the development of emotional awareness and reflective thinking into curricula may therefore foster resilience and academic success. Further longitudinal research is needed to explore causal mechanisms and intervention efficacy in broader academic contexts.

## 1. Introduction

Education serves as a platform for intellectual growth, personal development, and participative citizenship. The interconnected development of cognitive, social, and emotional skills should occur throughout the education years [1]. Competencies such as self-confidence, emotional regulation, and interpersonal skills are integral to achieving balance and fulfillment in academic settings. Psychological well-being (PWB) plays an important role in shaping students’ academic experiences and overall quality of life, with factors such as institutional support, family and peer relationships, and personal resilience strongly influencing PWB [2,3,4,5]. Students experiencing positive well-being, driven by internal motivation factors (e.g., personal interest and enjoyment), demonstrate higher academic performance, which reinforces their overall well-being [6,7,8]. Positive psychological states are associated with better academic performance, reduced mental health challenges, and greater engagement with learning environments [7,9,10,11].

The mental well-being of students in higher education institutions (HEIs) has emerged as a pressing global concern, with rising rates of anxiety, depression, and burnout reported across diverse academic contexts [12]. The transition to higher education is associated with academic pressures, financial stressors, and social isolation [13] that often exacerbate vulnerabilities to mental health challenges [14]. Recent epidemiological data suggest prevalence rates of depression, anxiety, and stress ranging from 27% to 53% across different academic institutions [15,16,17,18,19]. Mental well-being is a foundational prerequisite for academic success [10,20], social integration [20,21], and long-term career development [21,22]. The World Health Organization recognizes mental health challenges among young adults as a significant public health priority, emphasizing the need for evidence-based interventions within educational settings [12].

HEIs are increasingly acknowledging their responsibility in fostering environments conducive to mental well-being. While institutional responses have traditionally prioritized reactive measures such as counseling services, which have proven insufficient to address the scale and complexity of the issue [2,23], growing evidence advocates for proactive, skill-based interventions that empower students to navigate adversity [24,25] and manage academic stress [26,27]. Mental health promotion strategies must be embedded within educational practices and pedagogical approaches, creating a holistic framework for student development [28,29,30,31]. However, the efficacy of many interventions remains to be proven [32].

Among different approaches, fostering a critical thinking disposition (CTd) has been posited as a transformative strategy to nurture mental well-being [33]. Critical thinking transcends mere cognitive skills; it reflects a habitual willingness to engage with complexity, challenge assumptions, and regulate emotional responses during problem-solving [34]. CTd buffers against cognitive distortions (e.g., catastrophizing academic failure) and enhance adaptive coping mechanisms, thereby reducing psychological distress [35].

CTd is a mindset characterized by curiosity, analytical reasoning, systematic inquiry, truth-seeking, open-mindedness, and cognitive maturity [36], and may serve as a cognitive resource that empowers students to process complex emotional experiences, challenge maladaptive thought patterns, and develop adaptive coping strategies [37]. These dispositions may function as psychological buffers against academic stressors by enabling students to reframe challenges as manageable [33,38], employ evidence-based decision-making [39,40], and resist impulsivity—skills that align with emotional regulation and self-efficacy [36]. Metacognitive theory proposes that the reflective aspects of critical thinking (CT) could enhance emotional awareness and regulation [41], while self-determination theory indicates that the autonomy facilitated by critical thinking may satisfy fundamental psychological needs, thereby promoting psychological flourishing [42].

Empirical studies corroborate links between CTd and emotional intelligence [43,44,45,46] and mental wellbeing [47]. Research demonstrates that students with higher levels of critical thinking evidence better stress management [48], improved academic success [49], reduced procrastination [50], and protection against adverse life events in college students [33]. CTd training programs reduce the academic stress levels [51] and improve life satisfaction [52], significantly reduced anxiety [53] and improved resilience among university students [54]. Programs incorporating critical thinking, problem-solving, and metacognition significantly increase academic resilience, helping students better cope with stress and adversity [55,56] and thus contributing to students’ well-being.

However, comprehensive investigations examining the relationship between multidimensional constructs of CTd and psychological wellbeing remain notably scarce [57]. Gaps persist in understanding how CTd interacts with contextual factors (e.g., age, gender, academic field and study program) and specific dimensions of mental well-being (e.g., existential purpose, social connectedness).

Despite growing interest in the links between critical thinking and mental health, empirical studies simultaneously examining multidimensional critical thinking dispositions and psychological well-being remain scarce, particularly within European and Portuguese higher education contexts. Moreover, limited attention has been given to how specific dispositions may exert differential, or even contrasting, associations with distinct dimensions of well-being. Addressing these gaps, the present study provides a fine-grained analysis of critical thinking dispositions as predictors of psychological well-being in a multidisciplinary higher education sample.

The following research question was defined: What is the relationship between critical thinking dispositions and psychological well-being among higher education students? The study aims to examine associations between critical thinking dispositions and psychological well-being among undergraduate and graduate students across multiple disciplinary areas, as well as to identify potential modulatory effects of sociodemographic factors. Specifically, this study sought to investigate whether significant associations exist between critical thinking dispositions and psychological well-being among higher education students, and to determine which specific dimensions of critical thinking dispositions significantly predict psychological well-being after controlling for sociodemographic variables. Additionally, the study examined potential differences in both critical thinking dispositions and psychological well-being according to selected sociodemographic and academic characteristics.

By elucidating these relationships, we provide empirical foundations for educational approaches that simultaneously nurture cognitive capabilities and psychological resources, informing the development of integrated curricula that promote both academic excellence and mental health.

## 2. Materials and Methods

### 2.1. Design

This cross-sectional study aimed to investigate the relationship between critical thinking dispositions and psychological well-being in higher education students. This study was conducted from December 2024 to May 2025 using an online questionnaire available to participants on the Microsoft Forms platform (Microsoft 365; Microsoft Corporation, Redmond, WA, USA).

### 2.2. Participants

For participant recruitment, a non-probability convenience sampling approach was employed, facilitating cost-efficient access to the target population. Invitations were distributed via institutional email to students in higher education institutions (HEI) in Portugal. Eligibility criteria included being 18 years of age or older, current enrollment in any HEI, and Portuguese language proficiency.

The sample size was determined based on a 5% margin of error, balancing precision with feasibility, and a 95% confidence level, a standard threshold to minimize Type I errors [58]. Based on a population of 448,000 students in the country, the RAOSOFT Sample Size Calculator (Raosoft, Inc., Seattle, WA, USA) [59] indicated a required sample of 384 participants [60]. Although the total number of higher education students is provided for contextual purposes, the data for this study were collected using non-probability convenience sampling, thereby limiting the generalizability of the results. The achieved sample size (N = 429) exceeds the minimum recommended threshold and provides adequate statistical power (>0.80) to detect small-to-moderate effect sizes in correlational and regression analyses.

### 2.3. Instruments

Data collection involved the application of a sociodemographic questionnaire, which gathered information on participants’ age, gender, academic field, educational attainment (including associate’s, bachelor’s, master’s, and doctoral degrees), current academic year, and status as a working student (yes/no). Additionally, the study employed the Critical Thinking Dispositions Scale [60] and the Psychological Well-Being Scale [61,62].

The Critical Thinking Dispositions Scale (CTDS) is a self-report measure composed of 35 Likert-type items designed to evaluate seven key critical thinking dispositions: Truth-seeking involves a genuine eagerness to discover the truth and maintain objectivity when posing question; Open-mindedness reflects a willingness to accept and consider differing perspectives; Analyticity is the tendency to recognize potential problems, anticipate outcomes, and evaluate consequences, while systematicity emphasizes an organized, methodical, and focused approach to inquiry; Self-confidence relates to the degree of trust an individual has in their own reasoning abilities. Inquisitiveness represents intellectual curiosity and a desire for learning. Lastly, cognitive maturity reflects a person’s ability to make thoughtful and reflective judgments.

This scale was developed and validated for the Portuguese population by Lopes [60] using a sample of undergraduate and graduate students from Agricultural and Veterinary Sciences, Humanities and Social Sciences, Science and Technology, and Life and Environmental Sciences. The CTDS demonstrated excellent overall internal consistency (α = 0.94) and acceptable reliability across its seven subscales (α = 0.62–0.75). Scores on the CTDS range from 70 to 350 points, with each disposition contributing between 10 and 50 points. According to the interpretative guidelines proposed by Lopes et al., a total score of 280 or higher reflects a strong disposition toward critical thinking; scores between 210 and 279.9 indicate a positive disposition; scores from 140 to 209.9 denote ambivalence; and scores below 140 represent a low disposition. Corresponding subscale cut-off points are 39 for strong, 30 for positive, and 20 for ambivalent dispositions [60].

The PWBS, developed by Boylan e Ryff [61] and validated for the Portuguese population by Freire et al. [62], is designed to assess psychological well-being by evaluating students’ perceptions of their circumstances and their sense of success across various areas of life and personal growth. It examines well-being through six key dimensions: self-acceptance—the level of understanding and acceptance they had of themselves, including recognition of their own limitations (α = 0.82), positive relationships with others—the strength and depth of their connections with important others (α = 0.74), environmental mastery—how effectively they managed and adapted to their life circumstances (α = 0.77), autonomy—how closely they believed they were living according to their own values and convictions (α = 0.72), purpose in life—the extent to which individuals felt their lives had meaning and direction (α = 0.80), and personal growth—the degree to which they were utilizing their talents and realizing their potential (α = 0.71). The scale includes 42 items, rated on a Likert scale ranging from 1 (strongly disagree) to 6 (strongly agree).

Although widely used in health education research, both instruments assess transversal cognitive and psychological constructs applicable across disciplinary contexts, supporting their use in multidisciplinary higher education populations.

### 2.4. Procedures

Prior to data collection, ethical clearance was secured from the Ethics Committee of the Higher School of Health in the Lisbon area (Approval N° 11/2024; date of approval: 12 November 2024). Informed consent was obtained from all participants electronically before they accessed the online questionnaires.

The dissemination of the online questionnaire occurred in December 2024 through the official email channels of students enrolled in several HEI. The initial part of the online form provided a comprehensive overview of the research and facilitated the informed consent process. Participants proceeded to complete the series of scales, which took an average of 18 min. To prevent duplicate responses, the survey platform was configured to restrict multiple submissions from the same institutional account and IP address. The raw data were then exported to an Excel spreadsheet (Microsoft Excel, Microsoft 365; Microsoft Corporation, Redmond, WA, USA) and transformed into numerical codes.

### 2.5. Statistical Analysis

All statistical analyses were conducted using IBM^®^ SPSS^®^ Statistics version 29.0 for Windows (IBM Corp., Armonk, NY, USA). Descriptive statistics included means (M), standard deviations (SD), minimum (Min) and maximum (Max) values for continuous variables, and absolute and relative frequencies (n, %) for categorical variables. Prior to inferential analyses, normality was assessed using the Kolmogorov–Smirnov and Shapiro–Wilk tests and inspection of normal Q–Q plots, and homogeneity of variances was assessed using Levene’s test. Independent-samples *t*-tests and one-way analyses of variance (ANOVA) were used to examine group differences; when applicable, post hoc comparisons were performed using Tukey HSD, Gabriel, or Games–Howell tests, as appropriate. Pearson’s correlation coefficients were computed to examine bivariate associations among study variables. No missing data was observed for the variables included in the inferential analyses; therefore, all analyses were conducted on complete cases (N = 429). A sensitivity power analysis based on the achieved sample size (N = 429) indicated that, at α = 0.05 (two-tailed), the study had >80% power to detect correlations of r ≥ 0.15. For one-way ANOVA, effect sizes were estimated using eta-squared (η^2^), and for regression models the explained variance was reported using R^2^ and ΔR^2^.

Hierarchical multiple linear regression was conducted to identify predictors of psychological well-being. In the first step, sociodemographic variables (gender, age, year of study, and working-student status) were entered. In the second step, the seven critical thinking dispositions were included in the model. Regression assumptions were evaluated using standard residual diagnostics. Multicollinearity was assessed using variance inflation factor (VIF) and tolerance (VIF range: 1.057–2.054; tolerance range: 0.487–0.946). Statistical significance was set at *p* < 0.05 (two-tailed).

## 3. Results

### 3.1. Sample Description

The sample consisted of 429 higher education students with ages ranging from 18 to 61 years (M = 24.5; SD = 8.45). Table 1 presents the sociodemographic and academic characteristics of the participants.

Age groups were organized into four developmental categories based on stages of academic and psychosocial development in young adulthood [63]. transition and adaptation (18–21 years), initial consolidation (22–25 years), stabilization and growth (26–35 years), and maturity and experience (≥36 years). Approximately 29.8% of participants were working students, a group facing greater adaptive demands in managing well-being.

**Table 1:** Sociodemographic and Academic Characteristics of the Sample (N = 429).

Variable	Category	n	%
Gender	Male	69	16.1
	Female	351	81.8
	Other	9	2.1
Age (years)	Minimum = 18	Maximum = 61	Mean = 24.5
Age group	18–21 (Transition and adaptation)	227	52.9
	22–25 (Initial consolidation)	101	23.5
	26–35 (Stabilization and growth)	50	11.7
	≥36 (Maturity and experience)	51	11.9
Study cycle	Bachelor’s degree	383	89.3
	Master’s degree	46	10.7
Course	Occupational Therapy	43	10.0
	Nursing	188	43.8
	Veterinary Medicine	111	25.9
	Others	87	20.3
Year of study	1st year (Initial stage)	125	29.1
	2nd–3rd years (Intermediate stage)	193	45.0
	4th–6th years (Final stage)	111	25.9
Working student	Yes	128	29.8
	No	301	70.2

Note. SD = standard deviation. Percentages were calculated based on the total number of valid responses.

### 3.2. Description of Results for CTDS and PWBS (N = 429)

Table 2 presents descriptive statistics for the Critical Thinking Disposition Scale (CTDS) and the Psychological Well-Being Scale (PWBS), characterizing participants’ dispositional and emotional profiles.

### 3.3. Comparisons Between CTDS, PWBS, and Sociodemographic/Academic Variables

Independent samples *t*-tests examined differences in CTDS and PWBS scores according to key sociodemographic variables (Table 3). No gender differences emerged for either scale. However, working students scored significantly higher on both CTDS (t(427) = 2.24; *p* = 0.026; ΔM = 6.17) and PWBS (t(427) = 3.92; *p* < 0.001; ΔM = 13.26). Regarding study cycle, master’s students showed significantly higher PWBS scores compared to bachelor’s students (t(50.22) = −3.19; *p* = 0.002; ΔM = −14.35), though no difference was observed for CTDS.

One-way ANOVA analyses (Table 4) indicated that PWBS scores differed by age group (F(3.425) = 5.695; *p* = 0.001; η^2^ = 0.039), with students ≥36 years reporting the highest mean (M = 261.86). CTDS did not differ significantly across age groups (F(3.425) = 1.044; *p* = 0.373; η^2^ = 0.007). CTDS increased across years of study (F(2.426) = 7.020; *p* = 0.001; η^2^ = 0.032), from first year (M = 251.38) to final years (M = 263.09), whereas PWBS did not vary by year of study (F(2.426) = 1.244; *p* = 0.289; η^2^ = 0.006). Significant course differences emerged for PWBS (F(3.425) = 4.037; *p* = 0.008; η^2^ = 0.028), with Veterinary Medicine students reporting lower well-being (M = 194.27) than Nursing (M = 207.16) and Occupational Therapy students (M = 206.98); CTDS did not vary by course (F(3.425) = 1.045; *p* = 0.372; η^2^ = 0.007).

### 3.4. Correlation Between Critical Thinking Dispositions, Psychological Well-Being, Year of Study, and Age

Pearson correlations (Table 5) revealed positive associations between CTDS and PWBS dimensions (*p* < 0.05 or *p* < 0.01), ranging from weak to moderate in magnitude [64]. Total CTDS correlated most strongly with Personal Growth (r = 0.404; *p* < 0.01), Purpose in Life (r = 0.358; *p* < 0.01), Autonomy (r = 0.314; *p* < 0.01), and Environmental Mastery (r = 0.310; *p* < 0.01).

### 3.5. Predictors of Psychological Well-Being

Hierarchical multiple regression examined critical thinking dispositions as predictors of psychological well-being (Table 6). Model 1, containing sociodemographic variables (gender, age, year of study, working-student status), was significant (F(4.424) = 6.155, *p* < 0.001; R^2^ = 0.055), with age emerging as a positive predictor (β = 0.13; *p* = 0.021).

Model 2, adding the seven CTDS dimensions, significantly increased explained variance to 28.7% (R^2^ = 0.287; ΔR^2^ = 0.232; F(change) (7.417) = 19.406; *p* < 0.001). No evidence of multicollinearity was observed among the predictors (VIF range: 1.057–2.054; tolerance range: 0.487–0.946). Confidence in Reasoning (β = 0.27; *p* < 0.001), Cognitive Maturity (β = 0.24; *p* < 0.001), and Open-Mindedness (β = 0.15; *p* = 0.007) emerged as positive predictors. Notably, Inquisitiveness showed a negative effect (β = −0.15; *p* = 0.015) when controlling for other dispositions, suggesting a suppression pattern warranting further investigation. Although Inquisitiveness was positively associated with total PWBS in bivariate analyses (r = 0.209, *p* < 0.01; Table 5), it showed a negative adjusted association in the multivariable model (β = −0.15, *p* = 0.015; Table 6), consistent with a suppression pattern.

**Table 6:** Hierarchical multiple regression predicting total PWBS.

Model	Variable	B	SE B	β	t	*p*	R^2^ Change (ΔR^2^)	Sig. F Change
I	(Constant)	186.85	9.19	—	20.32	<0.001	0.055	0.001
	Gender	8.17	4.20	0.09	1.95	0.052		
	Age (years)	0.52	0.22	0.13	2.32	0.021		
	Year of Study	0.81	2.16	0.02	0.38	0.707		
	Working Student	−7.69	4.03	−0.11	−1.91	0.057		
II	(Constant)	44.63	16.06	—	2.78	0.006	0.232	0.001
	Gender	9.15	3.80	0.10	2.41	0.017		
	Age	0.57	0.20	0.15	2.88	0.004		
	Year of Study	−1.10	1.96	−0.03	−0.56	0.574		
	Working Student	−5.49	3.59	−0.08	−1.53	0.127		
	Truth-seeking	0.17	0.32	0.03	0.54	0.593		
	Open-mindedness	0.71	0.26	0.15	2.71	0.007		
	Analyticity	0.27	0.34	0.04	0.81	0.418		
	Systematicity	0.51	0.40	0.08	1.29	0.198		
	Confidence in Reasoning	1.72	0.31	0.27	5.49	< 0.001		
	Inquisitiveness	−0.92	0.38	−0.15	−2.44	0.015		
	Cognitive Maturity	1.42	0.35	0.24	4.05	< 0.001		

Note. Dependent variable: Total PWBS. *p* < 0.05; *p* < 0.01. B = unstandardized coefficient; SE = standard error; β = standardized coefficient; ΔR^2^ = change in the coefficient of determination; “—” indicates not applicable (ΔR^2^ and Sig. F Change are reported at the model/step level only)

## 4. Discussion

This study examined the relationship between critical thinking dispositions (CTD) and psychological well-being (PWB) among higher education students, exploring how these variables contribute to positive mental health. The findings should be interpreted considering the study’s correlational and cross-sectional design. While significant associations were identified between critical thinking dispositions and psychological well-being, these results do not permit causal inferences regarding directionality or underlying mechanisms. Accordingly, interpretations throughout this section focus on associations and potential explanatory pathways and are grounded in existing theoretical and empirical literature.

To provide context for interpreting the main findings, we first examine the demographic characteristics of the sample. The findings revealed a predominantly female sample (81.8%), with a mean age of 24.5 years, and primarily undergraduate students (89.3%). This distribution aligns with national and European trends in health-related fields, which are traditionally feminized [65,66], reflecting the growing presence of women in caring professions—roles that entail emotional implications associated with empathy and affective demands [67]. Despite the wide age range (18–61 years), over half of the participants were aged 18–21, a developmental period marked by identity and academic transitions that increase psychological vulnerability [68], which was reflected in lower scores on the Psychological Well-Being Scale. Older students, however, demonstrated higher levels of well-being, consistent with evidence suggesting that emotional maturity and social role integration foster affective stability and psychological balance [68].

Beyond these demographic patterns, the overall descriptive findings reveal important insights into students’ cognitive and emotional profiles. The average scores revealed generally favorable cognitive and emotional predispositions, with moderate to high global values on both the CTDS (M = 258.48) and the PWBS (M = 202.61). The dispositions of Systematicity and Inquisitiveness showed the highest mean values, indicating an organized, structured, and reflective approach to academic challenges. From a mental health perspective, this combination, encompassing curiosity, truth-seeking, and cognitive organization, supports resilience, self-efficacy, and perceived control, all of which are associated with lower anxiety and better adaptation [34,69]. Systematicity reflects an individual’s disposition to approach problems in an orderly and focused manner; individuals with high levels of this disposition tend to engage in comprehensive, reflective, and controlled thinking, which aligns with cognitive therapy principles aimed at shifting from automatic processing to more deliberate and reflective modes of thought [57].

Regarding the PWBS, Personal Growth and Purpose in Life emerged as the strongest dimensions, suggesting positive perceptions of progress and meaning. These are core indicators of positive mental health, consistent with Keyes’ model [69], which conceptualizes psychological well-being as the flourishing of human capacities. In contrast, lower means in Positive Relations and Environmental Mastery highlight potential vulnerabilities in social connectedness and environmental management, patterns commonly reported among health students exposed to demanding academic settings [14,70,71].

To further understand these patterns, we examined whether critical thinking and well-being profiles varied across sociodemographic groups. When comparing sociodemographic variables, no significant gender differences were found on either scale (CTDS or PWBS), suggesting that men and women share similar profiles of critical thinking and well-being. However, this finding should be interpreted with caution due to the predominance of female participants in the sample, which may have limited the sensitivity to detect gender-related effects. Working students scored higher on both scales, indicating that work experience may foster autonomy, structure, and a sense of competence, potentially acting as a protective factor for well-being [72]. In contrast, master’s students reported lower well-being levels, possibly due to increased academic and professional pressures associated with graduate-level demands [73].

The ANOVA results demonstrated that CTd increased with age and academic progression, supporting the notion that critical thinking is a developmental skill that matures with experience and education [36,74,75]. In contrast, the lack of significant differences in PWB across academic years suggests that emotional well-being tends to stabilize, relying more on personal and contextual factors than on academic advancement alone [76].

Having identified significant group differences, we then explored the interplay between specific critical thinking dispositions and well-being dimensions. Pearson correlations revealed consistent positive relationships between all CTd and dimensions of psychological well-being, with the strongest correlations observed for Personal Growth, Purpose in Life, Autonomy, and Environmental Mastery. These findings reinforce the view that critical thinking dispositions are not solely cognitive skills but also psychological resources that support emotional regulation and academic adjustment [34,77,78]. Cognitive Maturity and Confidence in Reasoning showed the most robust associations, underscoring the importance of reflective judgment, perspective-taking, and trust in one’s reasoning for mental health and adaptability [69,79].

To determine which specific dispositions independently predict well-being when considered simultaneously, hierarchical regression analysis was conducted. The hierarchical regression confirmed that, after controlling for sociodemographic variables, CTd explained 28.7% of the variance in psychological well-being. The dimensions Confidence in Reasoning, Cognitive Maturity, and Open-Mindedness were significant positive predictors. These findings align with theoretical frameworks suggest that the ability to reason confidently, consider multiple perspectives, and maintain cognitive flexibility enhances psychological well-being by promoting adaptive coping strategies and reducing cognitive distortions [57,80,81].

Although inquisitiveness is typically conceptualized as an adaptive cognitive disposition, its negative association with psychological well-being in the adjusted model was unexpected. Several hypothetical explanations may account for this pattern. Inquisitiveness reflects not only openness to learning but also a persistent drive for questioning and information seeking, which, within demanding, academically intensive contexts, may be experienced as cognitively taxing or emotionally destabilizing when not accompanied by effective regulatory strategies [82]. Importantly, emotional self-regulation and related constructs were not directly assessed in this study; therefore, such explanations should be regarded as theoretical hypotheses rather than empirically supported mechanisms. Future studies incorporating explicit measures of emotional regulation and stress appraisal are needed to test these possibilities.

Critical thinking dispositions, including open-mindedness, analyticity, and self-confidence, demonstrate positive and statistically significant associations with key dimensions of psychological well-being, namely mental health, autonomy, life purpose, and eudaimonic flourishing, confirming that critical thinking operates as a protective factor for mental health, fostering autonomy, resilience, and a sense of purpose [36,69,83]. Evidence indicates that this relationship is influenced by moderating variables such as mindfulness and by mediating mechanisms including impulsivity and cognitive distortions, which together shape the magnitude and direction of these effects [57,80,81].

Contextual variables such as institutional environment, teaching approaches, social support, and cultural background can substantially influence both CTd and well-being among higher education students. Research has shown that learning climates promoting autonomy, open dialogue, and reflective practices tend to enhance students’ critical engagement and psychological flourishing [84,85,86]. Moreover, highly competitive or rigid academic contexts may foster stress and reduce opportunities for critical reflection. Cultural dimensions also play a crucial role, as students’ conceptions of critical thinking and well-being vary across sociocultural settings [84,87]. Therefore, without controlling for or adequately accounting for such contextual influences, the observed associations between CTd and well-being may be partly confounded by institutional and cultural factors.

Despite these limitations, the findings carry important practical implications for higher education. Overall, the findings underscore the need for integrated pedagogical strategies that combine the development of critical thinking with mental health promotion, emotional literacy, and peer support initiatives. Implementing reflective curricula, emotional supervision, and brief psychotherapeutic interventions can strengthen both cognitive skills and psychological resources essential for well-being and academic success [88].

The results confirm that promoting critical thinking means promoting mental health: the ability to think clearly, reflect, and question constructively translates into greater emotional balance, resilience, and a sense of personal fulfillment, core elements of psychological flourishing in higher education students. Investing in pedagogical strategies that integrate reflective reasoning, emotional regulation, and psychological support offers a promising pathway toward educating more balanced, autonomous, and resilient students capable of facing personal, academic, and professional challenges with clarity and humanity.

## 5. Study Limitations

Several limitations should be acknowledged when interpreting these results. First, the cross-sectional design precludes conclusions about causality or temporal precedence between critical thinking dispositions and psychological well-being. Longitudinal studies are needed to determine whether changes in critical thinking dispositions precede, follow, or reciprocally interact with changes in well-being.

Second, all data were collected using self-report instruments, which may be subject to social desirability bias, common method variance, and individual differences in self-perception. The exclusive reliance on self-report measures also prevented the triangulation of findings with observational or performance-based assessments of critical thinking. Beyond methodological constraints, the sample composition presents important considerations for generalizability.

Third, data collection relied on an online survey format, which may have introduced selection bias by favoring students more comfortable with digital participation or with greater availability. Fourth, although participants were drawn from multiple higher education institutions, the sample was predominantly female and largely composed of students from health-related programs, limiting the generalizability of the findings to other academic fields and more gender-balanced populations. However, this gender distribution aligns with the existing gender distribution in higher education programs in the fields of Health Sciences, Social Sciences, and Economics. The restriction to health-related programs further limits the extent to which these findings can be extrapolated to students in science, technology, engineering, or humanities disciplines.

Finally, contextual factors specific to the study setting warrant consideration. Cultural and institutional factors specific to the Portuguese higher education context may have influenced both critical thinking dispositions and perceptions of psychological well-being, suggesting caution when extrapolating these results to other educational or cultural settings.

Despite these limitations, the study provides a nuanced examination of the multidimensional relationship between critical thinking dispositions and psychological well-being, offering a robust foundation for future longitudinal and intervention-based research.

## 6. Conclusions

Within the limits of a cross-sectional design and a predominantly health-related student sample, this study provides evidence of consistent associations between critical thinking dispositions and psychological well-being in higher education students. Confidence in reasoning, cognitive maturity, and open-mindedness emerged as particularly salient correlates of well-being, underscoring the relevance of reflective and flexible thinking dispositions in academic contexts.

These findings also reveal important developmental patterns. The study showed that age and academic experience are positively related to higher levels of critical thinking and psychological well-being, suggesting that these competencies may develop through a combination of personal maturity and formative educational experiences. The unexpected pattern observed for inquisitiveness suggests that not all critical thinking dispositions relate to psychological well-being in uniform ways, highlighting the need to examine contextual and individual moderators rather than assuming uniformly protective effects. This finding also underscores the importance of examining how curiosity interacts with broader psychosocial and contextual factors.

These findings have practical implications for higher education. They underscore the relevance of integrating critical thinking development—particularly dispositions related to confidence in reasoning, cognitive maturity, and open-mindedness, into student well-being promotion strategies. However, given the cross-sectional design, these implications should be interpreted as associational and warrant confirmation in longitudinal and intervention studies.

From an applied perspective, higher education institutions may consider curricula and student support structures that foster reflective and ethical reasoning alongside socioemotional competence-building and accessible psychological support services. Pedagogical approaches such as mentoring, problem-based and experiential learning, and structured reflective activities represent promising avenues for supporting both cognitive and socioemotional development. Complementary interventions, including mindfulness training and emotional literacy workshops may further enhance students’ socioemotional skills and psychological well-being. These findings point to important directions for future research, particularly supporting further investigation into integrated educational approaches that address both cognitive dispositions and psychological well-being through longitudinal and intervention-based research designs.

## Figures and Tables

**Table 2:** Descriptive Statistics for CTDS and PWBS Dimensions (N = 429).

CTDS	Item No.	Min./Max	M	SD	Average Score (M/Total Items)
Truth-seeking	4	20–50	34.74	5.57	8.69
Open-mindedness	4	15–50	37.17	6.87	9.29
Analyticity	4	10–50	36.99	5.23	9.25
Systematicity	7	10–50	38.39	4.82	5.48
Confidence in reasoning	5	10–50	36.73	5.17	7.35
Inquisitiveness	7	21–50	37.69	5.37	5.38
Cognitive maturity	4	20–50	36.73	5.43	9.18
Total CTDS	35	141–329	258.48	26.21	7.39
**PWBS**	**Item No.**	**Min./Max**	**M**	**SD**	**Average Score (M/Total Items)**
Autonomy	7	12–48	32.70	6.71	4.67
Environmental mastery	7	9–48	31.46	6.90	4.49
Personal growth	7	22–48	38.64	6.28	5.52
Positive relationships with others	7	14–42	30.06	6.14	4.29
Purpose in life	7	18–49	37.41	6.86	5.34
Self-acceptance	7	8–49	32.33	8.81	4.62
Total PWBS	42	96–276	202.61	32.59	4.82

Abbreviations: M = Mean; SD = Standard deviation.

**Table 3:** Independent Samples *t*-Tests for CTDS and PWBS by Sociodemographic Variables (N = 429).

	CTDS				PWBS			
Variables	*t*(*df*)	*p*	Mean Diff (ΔM)	95% CI [Lower; Upper]	*t*(*df*)	*p*	Mean Diff (ΔM)	95% CI [Lower; Upper]
Gender	−0.64 (427)	0.521	−2.21	[−8.99; 4.56]	−1.71 (427)	0.088	−7.30	[−15.70; 1.10]
Working student	2.24 (427)	0.026	6.17	[0.76; 11.58]	3.92 (427)	< 0.001	13.26	[6.61; 19.90]
Study cycle	−1.11 (52.20)	0.270	−3.76	[−10.54; 3.01]	−3.19 (50.22)	0.002	−14.35	[−23.38; −5.31]

Note. *p* < 0.05 indicates statistical significance.; CI = confidence interval. Data analyzed using two-tailed independent samples *t*-tests.

**Table 4:** One-Way ANOVA according to Age Group and Academic Variables.

Academic Variable	CTDS M (SD)	PWBS M (SD)
**Age group**		
18–21 → Transition and adaptation	256.49 (24.94)	198.14 (32.17)
22–25 → Initial consolidation	259.61 (25.39)	201.69 (32.24)
26–35 → Stabilization and growth	261.76 (33.22)	210.56 (34.04)
≥36 → Maturity and experience	261.86 (25.61)	216.47 (29.08)
ANOVA *	F = 1.044; *p* = 0.373; η^2^ = 0.007	F = 5.695; *p* = 0.001; η^2^ = 0.039
**Course**		
Occupational Therapy	263.32 (30.31)	206.98 (38.84)
Nursing	259.18 (27.99)	207.16 (32.43)
Veterinary Medicine	258.13 (21.51)	194.27 (29.96)
Other	255.02 (25.51)	201.24 (31.11)
ANOVA *	F = 1.045; *p* = 0.372; η^2^ = 0.007	F = 4.037; *p* = 0.008; η^2^ = 0.028
**Year of study**		
1st year—Initial	251.38 (27.53)	199.27 (34.05)
2nd–3rd years—Intermediate	260.42 (23.35)	202.84 (32.33)
4th–6th years—Final	263.09 (27.97)	205.95 (31.26)
ANOVA *	F = 7.020; *p* = 0.001; η^2^ = 0.032	F = 1.244; *p* = 0.289; η^2^ = 0.006

* One-way ANOVA; post hoc comparisons were performed using Tukey HSD, Gabriel, or Games–Howell tests, as appropriate (α = 0.05).

**Table 5:** Pearson Correlations between CTDS and PWBS, Year of Study, and Age.

Variables	Autonomy	Environmental Mastery	Personal Growth	Positive Relations	Purpose in Life	Self-Acceptance	Total PWBS	Age	Year of Study
Truth-Seeking	0.134 **	0.138 **	0.263 **	0.190 **	0.273 **	0.035	0.210 **	0.120 *	0.145 **
Open-mindedness	0.128 **	0.142 **	0.249 **	0.178 **	0.147 **	0.128 **	0.203 **	−0.009	0.213 **
Analyticity	0.262 **	0.178 **	0.260 **	0.187 **	0.218 **	0.201 **	0.277 **	0.051	0.034
Systematicity	0.242 **	0.273 **	0.252 **	0.169 **	0.357 **	0.273 **	0.337 **	0.018	−0.024
Confidence in Reasoning	0.353 **	0.283 **	0.299 **	0.170 **	0.238 **	0.377 **	0.374 **	0.021	−0.037
Inquisitiveness	0.124 **	0.138 **	0.293 **	0.192 **	0.193 **	0.076	0.209 **	0.089	0.147 **
Cognitive Maturity	0.295 **	0.353 **	0.318 **	0.266 **	0.314 **	0.313 **	0.397 **	0.083	0.167 **
Total CTDS	0.314 **	0.310 **	0.404 **	0.284 **	0.358 **	0.287 **	0.414 **	0.076	0.146 **
Year of Study	0.050	0.104 *	0.111 *	0.086	−0.005	0.064	0.086	0.275 **	-
Age	0.182 **	0.174 **	0.217 **	0.124 *	0.071	0.138 **	0.192 **	-	-

Note. *p* < 0.01 (**); *p* < 0.05 (*).

## Data Availability

Due to GDPR requirements and the confidentiality conditions described in the informed consent, the individual-level data are not publicly available. A de-identified minimal dataset can be provided to the Editors for internal review upon request.

## Abbreviations

The following abbreviations are used in this manuscript:

CT	Critical Thinking
CTd	Critical Thinking Dispositions
CTDS	Critical Thinking Dispositions Scale
HEI/HEIs	Higher Education Institution(s)
PWB	Psychological Well-Being
PWBS	Psychological Well-Being Scale
